# Pulse wave velocity demonstrates increased aortic stiffness in newly diagnosed, antiretroviral naïve HIV infected adults: A case-control study

**DOI:** 10.1097/MD.0000000000029721

**Published:** 2022-08-26

**Authors:** Pieter-Paul S. Robbertse, Anton F. Doubell, Steve Innes, Carl J. Lombard, Philip G. Herbst

**Affiliations:** a Division of Cardiology, Department of Medicine, Faculty of Medicine and Health Sciences, Stellenbosch University and Tygerberg Hospital, South Africa; b University of Pittsburgh HIV-Comorbidities Research Training Programme in South Africa; c Department of Paediatrics and Child Health, Family Centre for Research with Ubuntu (FAMCRU), Stellenbosch University, South Africa; d Desmond Tutu HIV Centre, University of Cape Town, South Africa; e Biostatistics Unit, South African Medical Research Council, South Africa; f Division of Epidemiology and Biostatistics, Department of Global Health, Stellenbosch University, South Africa.

**Keywords:** aortic stiffness, arterial stiffness, carotid-femoral pulse wave velocity, HIV-associated cardiovascular disease

## Abstract

Increased aortic stiffness is an important predictor of cardiovascular disease (CVD). It remains controversial whether HIV infected persons have increased aortic stiffness at the time of HIV diagnosis. An explorative, case-control study was performed using carotid-femoral pulse wave velocity (PWV) in a newly diagnosed, antiretroviral treatment (ART)-naïve cohort with modest baseline cardiovascular risk.

We recruited 85 newly diagnosed adults without known CVD from health care facilities in South Africa (43 female; mean age 33). Median CD4 count was 285, IQR 156–393 cells/µL. Twenty two HIV uninfected controls were recruited from the same facilities (8 female; mean age 33). PWV was measured using the Vicorder module (Skidmore Medical, United Kingdom) using a corrective factor of 0.8.

The HIV infected group’s mean PWV measured 11% higher than controls (5.88 vs 5.28 m/s; *P* = .02). Median aortic distensibility in HIV infected persons was 18% lower than controls (0.37 vs 0.45 mm Hg^−1^; *P* = .009). Multivariate analysis revealed that the difference in PWV between groups remained significant when corrected for age, sex, mean blood pressure and kidney function (mean difference 0.52 m/s; *P* = .01). Mean blood pressure, estimated glomerular filtration rate, HIV infection per se, age and male sex were important associations with increased PWV.

Our study provides evidence for increased aortic stiffness in ART naïve adults already demonstrable at the time of HIV diagnosis. The cohort’s young age and recent HIV diagnosis makes atherosclerosis a less likely explanation for the difference. Alternative, potentially reversible, explanations that require further research include vasomotor tone abnormalities and endothelial dysfunction.

## 1. Introduction

Persons infected with Human immunodeficiency virus (HIV) are at increased risk for cardiovascular disease (CVD). In the era of antiretroviral therapy (ART), it is estimated that people living with HIV (PLWH) have almost double the risk of developing cardiac dysfunction.^[1]^ CVD in PLWH represents a spectrum of disorders that is different for persons on ART than those not on ART.^[2]^ Furthermore, contrasting disease profiles are encountered in developing and developed countries thought to be explained primarily by differences in the degree of immunosuppression.^[3]^ Although not fully understood, CVD appears to be driven by a combination of factors that work synergistically to cause pathology.^[3]^ Important mechanisms thought to drive pathology include immune activation and chronic inflammation, accelerated vascular aging, atherosclerosis, and coronary artery disease.^[2–5]^

Various technologies are available to study vascular abnormalities that mainly focus on the detection of established structural problems like atheromatous plaques, coronary artery calcium, and carotid intima-media thickening.^[6,7]^ However, it has been demonstrated that endothelial dysfunction precedes the development of atherosclerotic plaques.^[6]^ This functional abnormality is a marker of early coronary artery disease and appears to be the first stage in the development of atherosclerosis and its complications.^[6]^

Carotid-femoral pulse wave velocity (PWV) is a sensitive tool to assess early alterations in aortic stiffness and may be used to evaluate subclinical atherosclerosis.^[6,8]^ PWV is the current gold standard for noninvasive aortic stiffness quantification.^[9–12]^ PWV has been correlated with cardiovascular outcomes and is increasingly used as a surrogate end point for CVD.^[9,13]^ It is possible that factors other than atherosclerosis, including possible reversible factors, may contribute to elevated PWV.

Controversy remains as to whether PWV differs significantly between PLWH and HIV uninfected persons.^[7]^ Prior research studies aimed at answering this have used different study methodologies including methods of acquiring PWV, making comparison between studies difficult. Furthermore, many studies included heterogeneous populations including persons with varied cardiovascular risk profiles and cardiovascular comorbidities, as well as persons already on ART.^[7]^

We set out to evaluate aortic stiffness in a relatively young, newly diagnosed, ART-naïve cohort with modest baseline cardiovascular risk. PWV was used to compare arterial stiffness between healthy controls and a group of HIV infected persons before their initiation of ART. The driving factors of altered arterial stiffness were explored to further our understanding of the underlying pathophysiology and its long-term health implications.

## 2. Methods

### 2.1. Study design and participants

Outpatients naïve to ART (n = 85) were recruited from health facilities in the Western Cape, South Africa from January 2020 to July 2021. The majority of participants had a new diagnosis of HIV infection. The ART status of participants was confirmed on history and cross checked on a centralized pharmacy system. Healthy, HIV negative volunteers (n = 22) were recruited as a control group from the same healthcare facilities and surrounding areas. Inclusion criteria were age ≥ 18 and ≤ 55 years, no history or clinical features of congenital or acquired CVD (not including hypertension), and no acute intercurrent illness (including current coronavirus disease 2019). All participants recruited after March 2020 underwent SARS-CoV-2 PCR testing prior to enrollment. Ethical approval was granted by the Stellenbosch University Human Research Ethics Committee and signed informed consent was obtained from all participants.

### 2.2. Aortic stiffness

Aortic stiffness was measured in a temperature controlled room using the Vicorder module (software version 8.3.7698.18341; Skidmore Medical, United Kingdom). Aortic stiffness was expressed in terms of pulse wave velocity and aortic distensibility. PWV was calculated as [direct path length (L)/ transit time (TT) × 0.8] Aortic distensibility (D) was expressed according to the Bramwell-Hill equation as 3.57/PWV^2^.^[10]^ The direct measurement approach with a scaling factor of 0.8 was employed to standardize the measurements in accordance with current literature.^[14]^ This scaling factor allows for better comparison with cardiovascular magnetic resonance imaging (CMR) acquired PWV.^[14]^ All participants were instructed to withhold use of any caffeine or tobacco products on the day of the examination. Participants were placed in the supine position and allowed at least 10 minutes of rest. A 100 mm blood pressure cuff was placed around the upper thigh and a 30 mm partial cuff was placed around the neck at the level of the carotid arteries just above the level of the thyroid prominence. Direct path length was measured by the same operator from the suprasternal angle to the top of the femoral blood pressure cuff. Both cuffs were inflated simultaneously to 60 mm Hg to record the femoral and carotid waveforms. Continuous recordings were made on individual heart beats for at least 10 minutes. Automated software computed the foot of the wave using an intersecting tangent algorithm. The time delay between the foot of the carotid and femoral waveforms provided the averaged TT every 3 seconds. Data was screened in real time for motion artifacts and these segments were discarded. The TT dataset was used to determine the coefficient of variation (CV) for the individual. Datasets with a CV of <30% were judged to be of sufficient quality to be included in the final analysis. The mean TT of the recording was used to calculate PWV.

### 2.3. Anthropomorphic, clinical, and biochemical data

Assessments took place in the morning after a fast of at least 10 hours. All participants underwent a full consultation and physical examination by the principal investigator. Smoking and alcohol use was quantified from history. Smoking was classified as “ever”, “never,” or “current” and the number of current daily cigarettes was captured in accordance with contemporary literature.^[15]^ Duration of HIV infection was calculated from the first positive rapid or serological HIV test on the electronic database of the National Health Laboratory Service (NHLS). Manual blood pressure on the right arm was measured in the seated position after a 10-minute rest. The same wall-mounted sphygmomanometer unit (Welsch Allyn, New York) was used in all cases. A standard adult size cuff was employed. In larger persons, a large adult cuff was used to cover at least two-thirds of the person’s upper arm. Mean arterial pressure was calculated as [(1/3 × pulse pressure) + diastolic blood pressure].^[16]^ Height and weight were measured and used to calculate body mass index (BMI). Waist circumference was measured at the level of the umbilicus with the patient in the standing position. A 12-lead electrocardiogram (ECG) was performed by a central ECG laboratory to screen for underlying cardiovascular disease and used to evaluate the resting heart rate and rhythm. A 6-minute walk distance was performed using standardized methodology.^[17]^ Fasting blood samples were sent to the NHLS for measurement of urea and electrolytes, glucose, blood lipids, full blood count, differential cell count, HIV enzyme-linked immunosorbent assay, high sensitivity C-reactive protein (hsCRP), CD4 count and HIV RNA PCR viral load. Estimated glomerular filtration rate (eGFR) was calculated using the Cockcroft-Gault formula: eGFR = [(140 – age) × weight (kg)]/serum creatinine (µmol/l). A corrective factor of 0.85 was used if the participant was female. Metabolic syndrome was defined according to National Cholesterol Education Program (NCEP) adult treatment panel (ATP) III report.^[18]^

### 2.4. Statistical analysis

Statistical analyses were performed using SPSS version 27 (IBM corporation, New York) and STATA version 17.0 (Stata Corporation, USA). Continuous data are presented as mean ± standard deviation. For highly skewed data, the median and interquartile ranges are stated. Categorical data is presented as frequencies with percentages. Normality of data was tested using the Shapiro–Wilk test. Significant differences between the HIV infected and control group were determined using the chi-square test or the 2-sample t-test, as appropriate. The outcome variable aortic distensibility was log transformed for analysis. For other skewed data, the nonparametric Mann–Whitney U test was used. Spearman correlation coefficients (*r*_*s*_) were used to assess the strength and significance of correlations of PWV with other continuous variables in both study groups. To identify multivariate correlates of PWV and aortic distensibility, linear regression analyses were performed. A “basic confounders” linear regression model was created to adjust PWV for nonHIV related variables age, sex, and ethnicity. An “additional effects” model was constructed to assess the influence of additional variables that may be influenced by HIV infection or its’ sequelae. Using the above model’s results, a final model was created to assess predictors of PWV in the cohort. Statistical significance testing was 2-tailed and set at *P* ≤ .05.

## 3. Results

### 3.1. Baseline clinical characteristics

Participants with HIV infection shared similar characteristics with the control group and were well matched in terms of age and sex (Table 1). Despite a strong trend to more smokers in the HIV infected group (49% vs 27%) this difference did not reach statistical significance; *P* = .06. Both groups were relatively young with a mean age of 33 years. The HIV infected group had a lower median BMI (24 kg/m^2^ vs 29 kg/m^2^; *P* < .001), as well as smaller waist circumference (80 vs 96 cm; *P* < .001) compared to their uninfected counterparts. Two HIV positive participants had a known diagnosis of hypertension. One was well controlled and on amlodipine 10 mg/hydrochlorothiazide 12.5mg and the other was taking hydrochlorothiazide 12.5 mg previously. One control with hypertension was included to match the hypertensives in the HIV group. No persons in either study group were taking statins, nitrates, or hormone replacement therapy. Tuberculosis (TB) co-infection was frequently encountered. Sixteen percent of the HIV infected group was diagnosed with TB at baseline with sputum polymerase chain reaction (GeneXpert) and/or auramine staining. One participant was found to have incidental pulmonary TB based on radiographic findings. No TB-coinfection was present in the control group. One control had a prior diagnosis of mild coronavirus disease 2019.

**Table 1:** Baseline characteristics of the study population.

Parameter	HIV infected group (n = 85)	Control group (n = 22)	*P* value
Age, years	33 ± 8	33 ± 7	0.94
Female n, (%)	43 (51)	11 (50)	0.96
Ethnicity n, (%)			**0.001**
African	68 (80)	9 (41)	
Mixed race	16 (19)	12 (54)	
Caucasian	1 (1)	1 (5)	
BMI, kg/m^2^	24 (21–25)	29 (24–36)	**<0.001**
Waist circumference, cm	78 (74–85)	97 (81–113)	**<0.001**
Smoking status n, (%)			0.16
Ever	40 (47)	6 (27)	
Occasional	2 (2)	nil	
Never	43 (51)	16 (73)	
Daily cigarettes (in persons that smoke)	7 ± 8	4 ± 3	0.052
Current tuberculosis co-infection n, (%)	14 (16)	nil	**0.04**
Duration of known HIV infection, days	10 (5–20)	–	–
6-minute walk test, meters	610 ± 93	637 ± 84	0.2
Resting heart rate, beats/minute	74 ± 15	70 ± 10	0.3
Systolic blood pressure, mm Hg	112 ± 16	119 ± 13	0.08
Diastolic blood pressure, mm Hg	71 ± 10	75 ± 9	0.057
Mean arterial pressure, mm Hg	85 ± 12	90 ± 9	**0.05**
Pulse pressure, mm Hg	41 ± 10	43 ± 10	0.4
Serum creatinine	70 ± 15	72 ± 14	0.6
eGFR, ml/min	92 (78–100)	108 (80–127)	**0.016**
Fasting blood glucose, mmol/l	4.5 ± 0.6	5.0 ± 0.6	**0.002**
High-density lipoproteins, mmol/l	1.1 ± 0.4	1.3 ± 0.3	**0.001**
Low-density lipoproteins, mmol/l	2.0 ± 0.8	2.6 ± 0.9	**0.007**
Serum triglycerides, mmol/l	1.0 ± 0.4	1.1 ± 0.7	0.96
Triglyceride: high density lipoproteins ratio	1.1 ± 0.8	0.8 ± 0.7	0.2
hsCRP, ng/l	3.2 (0.8–23.3)	2.4 (1.8–9.23)	0.5
Hematocrit, %	39 ± 0.06	42 ± 0.04	**0.01**
CD4 count, cells/µL	285 (156–393)	−	−
Viral Load (log), copies/ml	4.98 (4.1–5.5)	−	−

Continuous variables are mean ± standard deviation or median (interquartile range) unless otherwise specified.

BMI = body mass index, eGFR = estimated glomerular filtration rate, hsCRP = high sensitivity C-reactive protein.

Metabolic syndrome was rarely encountered in both study groups. One control and 2 participants fulfilled NCEP ATP III criteria.

The eGFR, fasting blood glucose, high-density lipoproteins (HDL), low-density lipoproteins (LDL), and hematocrit were all significantly lower in the HIV infected group (*P* values < 0.01). Although the mean hsCRP in the HIV infected group was more than double that of the control group, the difference between the HIV infected and control groups did not reach statistical significance (5.2 vs 12.5 g/nl; *P* = .5). No difference was demonstrable between the 2 groups’ cardiopulmonary fitness as assessed by the 6-minute walk distance. The HIV infected group’s diastolic and mean blood pressures tended to be lower than controls. This neared, but did not reach statistical significance (*P* = .057 and *P* = .05). The median time from HIV diagnosis to the baseline visit was 10 days.

### 3.2. Aortic stiffness

Readings within the HIV and control group were of good quality with a mean CV of 4.5% and 3.6% respectively (Table 2). One female control was excluded from the final analyses due to the CV calculating to more than the prespecified 30% required for inclusion. The mean PWV was 0.6 m/s higher in HIV infected persons compared to controls (5.88 m/s vs 5.28 m/s; *P* = .02). This represents an 11% higher PWV in the HIV infected group. Median aortic distensibility was 18% lower in persons with HIV compared to controls (0.37 mm Hg^−1^ vs 0.45 mm Hg^−1^; *P* = .009).

**Table 2:** Aortic stiffness parameters of the study population.

Parameter	HIV infected group (n = 85)	Control group (n = 21)	Mean difference	Mean difference (95% confidence interval)	*P* value
**PWV**					
PWV, m/s	5.88 ± 1.0	5.28 ± 1.2	0.6	0.10 to 1.09	**0.02**
PWV without 0.8 scaling factor, m/s	7.35 ± 1.2	6.6 ± 1.5	0.75	0.13 to 1.36	**0.02**
PWV adjusted with basic confounders model, m/s	–	–	0.54	0.05 to 1.02	**0.03**
PWV adjusted with final model, m/s	–	–	0.52	0.11 to 0.94	**0.01**
**Aortic distensibility**					
Aortic	0.37	0.45	−0.11*	−0.18 to −0.03*	**0.009** *
distensibility, mm Hg-1	(IQR: 0.30 to 0.46)	(IQR: 0.35 to 0.54)			
Aortic distensibility adjusted with final model, mm Hg-1	–	–	−0.09*	−0.16 to −0.024*	**0.008** *
CV, %	4.6 ± 4	3.6 ± 1.7	–	–	0.3

Continuous variables are mean ± standard deviation or median (interquartile range) unless otherwise specified.

CV = coefficient of variability, IQR = Inter quartile range, PWV = Carotid-femoral pulse wave velocity.

* Log10 transformed data.

The TB prevalence differed significantly between the 2 study groups (14% vs 0%; *P* = .04). However, PWV did not differ significantly between HIV infected participants with and without TB-infection (5.92 ± 0.8 vs 5.87 ± 1.0 m/s; *P* = .9). Resting heart rate between HIV infected participants with and without TB-infection showed a trend towards difference. This did however not reach statistical significance (81 ± 16 vs 73 ± 15 beats/minute; *P* = .06).

### 3.3. Bivariate analysis

Some correlations between PWV and other variables were different within the HIV infected and control groups. Within the HIV infected group, mean blood pressure showed a negative correlation (r_s_ = −0.42; *P* < .001) and age a positive correlation (r_s_ = 0.37; *P* < .001) with PWV.

Within the control group, only heart rate (r_s_ = −0.51; *P* = .02) was negatively correlated with PWV. No correlation with age was demonstrable in the controls (r_s_ = 0.22; *P* = .3).

Neither group demonstrated a significant correlation between PWV and BMI, waist circumference, TB-infection, hs-CRP, fasting glucose, eGFR, HDL, LDL, and hematocrit (*P >* .2). No correlation was found between PWV and days of known HIV diagnosis (rs = −.09; *P* = .4), CD4 count (rs = −.012; *P* = .3), or HIV viral load (rs = −.09; P = .4).

### 3.4. Multivariate analysis

In the basic confounders linear regression model (Supplemental file A, http://links.lww.com/MD/G985), HIV remained associated with elevated PWV [unstandardized coefficient (B) = 0.54, 95% confidence interval (CI) for B: 0.05 to 1.02, *P* = .03] Age and female sex were both significant predictors of PWV (B = 0.05, *P* < .001 and B = −0.43, *P* = .2). The additional effects regression model assessed the influence of ethnicity, BMI, mean blood pressure, heart rate, fasting blood glucose, eGFR, hematocrit, LDL, hsCRP, current TB-infection, and smoking history (Supplemental file B, http://links.lww.com/MD/G985) on PWV. Some of these included parameters could have been influenced by HIV infection. Neither ethnicity, nor TB-infection was associated with differences in PWV (*P* = .2 and 0.2). A final model was created to account for relevant and significant variables identified using the additional effects model (Table 3 and supplemental file C, http://links.lww.com/MD/G985). This model included age, sex, mean blood pressure, eGFR, and HIV infection. HIV infection (B = 0.52, 95% CI: 0.11 to 0.94, *P* = .01), higher mean blood pressure (B = 0.04, 95% CI: 0.03 to 0.06, *P* < .001), male sex (B = 0.37, 95% CI: 0.69 to 0.05, *P* = .02), increased age (B = 0.03, 95% CI: 0.003 to 0.05, *P* = .02) and decreased eGFR (B = −0.02, 95% CI: −0.02 to −0.01, *P* < .001) were all predictors of increased PWV. eGFR and mean blood pressure were highly significant at the level of *P* < .001. Furthermore, they had the strongest effect on PWV in this model. The final model was repeated with aortic distensibility (log transformed) as outcome variable. Variables that predicted decreased aortic distensibility were identical to the final PWV model and showed better precision (Supplemental file D, http://links.lww.com/MD/G985).

**Table 3:** Final linear regression analysis with carotid-femoral pulse wave velocity as outcome variable.

	Unstandardized coefficients	Standardized coefficients	*P* value	95% confidence interval for B	
	B	beta		Lower bound	Upper bound
Constant	2.232		0.007	0.627	3.837
Age (years)	0.026	0.189	0.024	0.003	0.048
Female sex (yes)	−0.369	−0.177	0.024	−0.689	−0.049
**HIV infected (yes**)	**0.524**	**0.201**	**0.013**	**0.112**	**0.936**
Mean BP (mm Hg)	0.043	0.472	0.000	0.026	0.059
eGFR (ml/min)	−0.015	−0.397	0.000	−0.021	−0.008

BP = blood pressure, eGFR = estimated glomerular filtration rate.

## 4. Discussion

This cross-sectional study is the first to evaluate aortic stiffness in such a young, relatively healthy group of ART-naïve, HIV infected adults. We believe this study assists to better represent African patients from subSaharan Africa in the literature.

The key finding was increased aortic stiffness using noninvasive PWV already demonstrable at the time of HIV diagnosis. This represents the earliest time point at which vascular abnormalities may be diagnosed and illustrates that alterations in aortic stiffness occur early in HIV infection and in the absence of ART. The modest cardiovascular risk of the HIV group, limited confounding factors, and the small impact of covariates on PWV from the regression analysis all provide evidence that HIV infection per se drives vascular dysfunction. These findings are in keeping with the current thinking that HIV infection is an independent risk factor for vascular dysfunction. Furthermore, by directly or indirectly influencing blood pressure and kidney function, HIV may cause further alterations in PWV.

### 4.1. Mechanism

The most accepted haemodynamic model of the arterial system is the propagative model. ^[11]^ In this model, peripheral arteries may modulate the central arterial pressure wave by means of a process called pressure amplification. The stiffness of peripheral arteries is modulated by *vasomotor tone*. Vasomotor tone in turn is dependent on *endothelial function*, the *sympathetic nervous system*, and the *renin-angiotensin-aldosterone system* (RAAS).^[19–21]^

**Figure 1.**
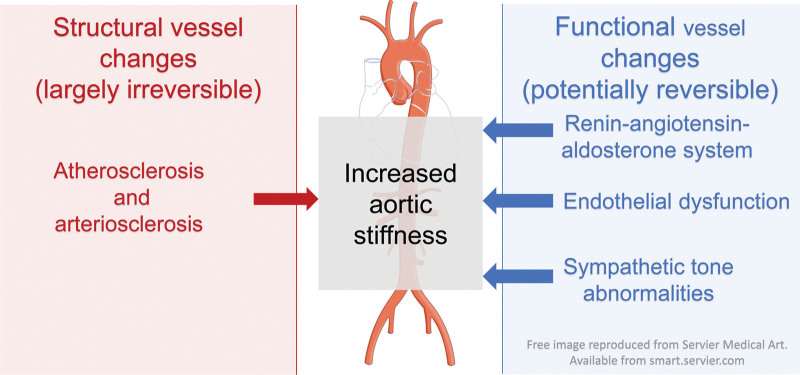
Factors influencing aortic stiffness.

Examining our findings, the increased aortic stiffness of the HIV infected group may, in part, be explained by functional vasomotor tone abnormalities, rather than established atherosclerosis (Fig. 1). Our cohort had a recent HIV diagnosis (median 10 days), is young, free from symptomatic CVD and largely absent risk factors that are associated with atherosclerosis. Smoking was not associated with elevated PWV in our analyses. Although the influence of subclinical atherosclerosis cannot be fully excluded, the above factors all make atherosclerosis a less likely explanation for the aortic stiffness findings. Arterial stiffness can be elevated in the absence of atherosclerosis and alternative mechanisms should be considered.^[22]^ We hypothesize that vasomotor tone abnormalities are responsible for the observed difference in PWV between our groups. In addition, the vasomotor abnormalities were initiated by HIV infection.

### 4.2. Vasomotor tone abnormalities

Our study was not specifically designed to assess the factors that underpin vasomotor tone, but some thoughts will be discussed in light of our findings.

The stiffness of peripheral arteries are influenced by the RAAS and can increase PWV through pressure amplification.^[10]^ In our HIV infected cohort, eGFR and mean blood pressure were highly significant predictors of PWV [standardized coefficient (β) =-0.41 and 0.47; *P* < .001] Furthermore, these parameters differ significantly between our 2 study groups (*P* = .016 and 0.05). Both these factors influence, and are in turn influenced by the RAAS, providing strong signal that the observed differences in PWV may have been facilitated by RAAS in our cohort. HIV has adverse effects on kidney function and blood pressure through various mechanisms, including direct viral effects on the kidney and vasculature.^[23]^ Additionally, excess mineralocorticoid signaling in the RAAS can lead to increased vascular and myocardial inflammation, abnormal collagen deposition, and decreased bioavailability of nitric oxide, causing vascular dysfunction.^[24]^

Although incompletely understood, endothelial dysfunction in HIV infected persons is well described and may be present in early HIV infection.^[25,26]^ Primary evaluation of endothelial function did not form part of our study and will require further investigation.

Using the resting heart rate as a crude gauge of sympathetic tone, no difference was noted between our study groups. Concordant with clinical experience, a large proportion of our HIV infected participants have concomitant TB-infection at the time of HIV diagnosis (16%). The heart rate between TB-infected and TB-negative participants of our HIV group did not differ significantly and would therefore not explain the differences in PWV. Investigations that specifically evaluate sympathetic tone would prove useful to study the interaction of sympathetic tone on aortic stiffness.

### 4.3. Significance and trajectory

Increased aortic stiffness using PWV is a well validated predictor of cardiovascular events and all-cause mortality.^[9,13]^ PWV’s ability to predict future CVD improves in younger individuals and groups with higher baseline risk. Additionally, PWV may assist to risk-stratify individuals who may benefit from more aggressive cardiovascular risk factor management more accurately.^[9,13]^ Based on meta-analysis data without the use of a scaling factor, it is estimated that for every 1 m/s increase in PWV, cardiovascular risk increases by about 14%.^[9]^

It may be argued that the PWV measurements of our HIV infected group are not grossly abnormal when compared to published normal reference ranges.^[14]^ However, significant difference at the time of HIV diagnosis is already evident when compared to our control group. When PWV without a scaling factor is used to compare our data, a 0.75 m/s difference is observed. Furthermore, these persons will spend the remainder of their lives with HIV infection. Even with ART and viral suppression, a state of chronic immune activation and systemic inflammation is inevitable.^[27]^ Together with the already present increase in aortic stiffness, cardiovascular risk will increase with age and the development of concomitant chronic diseases that are associated with age and chronic HIV infection.^[23]^

The treatment of HIV can increase cardiovascular risk as well. Although controversial, abacavir has been associated with increased risk of myocardial infarction in patients on ART.^[28]^ Studies on protease inhibitors have more consistently demonstrated deleterious effects on cardiovascular health by promoting pro-atherogenic lipid profiles and insulin resistance.^[28]^

The above factors all raise concern regarding the expected trajectory of the aortic stiffness abnormalities over time.

### 4.4. What can be done?

It is not known to what degree the aortic stiffness abnormalities are reversible with ART. Compared to our findings, research using CMR in patients already on ART has demonstrated a similar increase in aortic stiffness. Despite viral suppression, PWV was still 10% higher than controls in PLWH already on ART for a number of years.^[29]^ Bearing in mind that our study used the Vicorder module and not CMR, this figure is strikingly similar and may be an omen of ART’s limited long-term ability to normalize aortic stiffness. Limited data is available on ART’s ability to decrease aortic stiffness. Recent research evaluating biomarkers and PWV after ART initiation have provided some insight. Treatment with protease inhibitors was shown to decrease pro-atherogenic biomarkers in the short term, with a rebound increase later in treatment. Furthermore, the PWV remained largely unchanged at 6 months.^[30]^ The increased aortic stiffness detected at diagnosis in our study needs to be evaluated for reversibility in the mid- to long term. Newer ART’s used as current first line therapy have yet to be tested as a tool to improve aortic stiffness. This will add valuable insight into the underlying pathophysiology.

### 4.5. Study Limitations

This exploratory study was cross sectional in nature. Therefore, questions on causality cannot be directly answered. The need for prospective research is clear and will be of great importance to determine how PWV evolves after the initiation on ART. Furthermore, as the study was evaluating relatively healthy individuals at baseline, no outcome data is available that can be compared to PWV. In the absence of primary CVD outcomes, it may be of use to study surrogate CVD outcomes.

The recruitment of participants in the era of COVID-19 was challenging and significantly impacted the final number of participants. Our study would have benefited from a larger sample size to strengthen its statistical power and allow better generalization to our population. It is possible that a larger control group would have allowed us to prove differences between the group’s blood pressure readings, hsCRP, and smoking history with greater certainty.

Smoking was quantified using clinical history. The use of a cotinine assay would have provided a more accurate, but more costly assessment of the cohort’s smoking habits.

Even with meticulous history taking and contact tracing, estimating the duration of HIV infection remains a “best guess” scenario. We used the first documented HIV test as our reference for duration of infection as this can be determined with great certainty. Unfortunately, the true duration of HIV infection in our cohort is not known. Participants will have varying durations of untreated HIV infection before they present to health facilities. Late presentation is a phenomenon that is not unique to low- and middle income countries and is observed in the developed world as well.^[31]^ Based on the CD4 count, the overall level of immunosuppression in our cohort is significant with 65% of the cohort having a count of <350 cells/m^3^. This level of immunosuppression at presentation is not uncommon in our setting and in keeping with local data.^[32]^ The CD4 count may be used as an indication of the time since HIV seroconversion. However, its use is limited by various patient and disease factors that influence the rate of CD4 count decrease.^[33,34]^ This makes accurate estimation of the time since HIV seroconversion difficult. We believe our cohort is an accurate representation of the average new diagnosis of HIV in South Africa. PWW was measured using the Vicorder device, rather than using applanation tonometry that is frequently employed in PWV research. However, consensus has not been reached regarding the definitive method for measurement.^[35]^ Care has been taken to ensure the data is reliable and free from artifact. In addition, extended and averaged recordings were performed and screened by the same operator in real time. Any methodological or measurement error will apply to both study groups and will not affect the overall difference present between groups.

## 5. Conclusion

Our study demonstrates increased aortic stiffness in ART naïve adults at the time of HIV diagnosis. HIV infection per se, higher mean blood pressure, lower eGFR, higher age, and male sex were factors associated with increased aortic stiffness in our data. We hypothesize functional and potentially reversible mechanisms are responsible for this. Prospective research on the factors that underlie vasomotor tone are required. This will shed light on the underlying pathophysiology and may assist to develop preventative and screening strategies in a group of patients that currently suffer from disproportional CVD risk.

## Acknowledgments

The PROVE CVD study is supported in part by a Fogarty International Center HIV Research Training Program grant, National Institutes of Health, to the University of Pittsburgh and Stellenbosch University (D43TW010937). We thank Dr Ellen Ngarande for her assistance with patient logistics and data management.

## Author contributions

PS Robbertse was the primary investigator and responsible for the conception and design of the study, acquisition of data, analysis and interpretation of data, as well as drafting of the manuscript. PG Herbst, AF Doubell, S Innes and CJ Lombard contributed to the conception and design of the study, analysis and interpretation of data, as well as revising the manuscript critically for important intellectual content.

## Supplementary Material


