# Supplementary material for: Pulse wave velocity demonstrates increased aortic stiffness in newly diagnosed, antiretroviral naïve HIV infected adults: A case-control study
Source: Medicine (Baltimore). 2022 Aug 26;101(34):e29721. doi: 10.1097/MD.0000000000029721 (PMC9410660; doi:10.1097/MD.0000000000029721)
Supplement: Supplementary file 1 [file medi-101-e29721-s001.pdf]

Supplemental file A:

Crude linear regression analysis of PWV as outcome variable (dependant variable) and HIV infection as independent variable.

Model Summary

Model	R	R Square	Adjusted R Square	Std. Error of the Estimate
Crude Model	.228[a]	0.052	0.043	1.02213

a. Predictors: Constant, HIV infected (yes)

ANOVA[a]

	Sum of Squares	df	Mean Square	F	P value
Regression	5.958	1	5.958	5.703	.019[b]
Residual	108.655	104	1.045		
Total	114.612	105			

a. Dependent Variable: Carotid-femoral pulse wave velocity (m/s)

b. Predictors: Constant, HIV infected (yes)

Coefficients[a]

	Unstandardized Coefficients	Standardized Coefficients	P value	95% Confidence Interval for B	
	B	Beta		Lower Bound	Upper Bound
Constant	4.687		0.000	3.775	5.598
HIV infected (Yes)	0.595	0.228	0.019	0.101	1.089

a. Dependent Variable: Carotid-femoral pulse wave velocity (m/s)

Basic confounders linear regression model with PWV as outcome variable (dependant variable)

Model Summary

Model	R	R Square	Adjusted R Square	Std. Error of the Estimate
Basic confounders	.486[a]	0.236	0.206	0.93109

a. Predictors: Constant, African ethnicity (no), female sex (no), age (years), HIV infected (yes)

ANOVA[a]

	Sum of Squares	df	Mean Square	F	P value
Regression	27.053	4	6.763	7.801	.000[b]
Residual	87.559	101	0.867		
Total	114.612	105			

a. Dependent Variable: Carotid-femoral pulse wave velocity (m/s)

b. Predictors: Constant, African ethnicity (no), female sex (no), age (years), HIV infected (yes)

Coefficients[a]

	Unstandardized Coefficients	Standardized Coefficients		95% Confidence Interval for B	
	B	Beta	P value	Lower Bound	Upper Bound
Constant	3.858		0.000	2.385	5.331
Age (years)	0.051	0.371	0.000	0.027	0.074
Female sex (yes)	-0.426	-0.205	0.021	-0.785	-0.066
HIV infected (yes)	0.536	0.205	0.031	0.050	1.022
African ethnicity (no)	-0.075	-0.033	0.730	-0.506	0.355

a. Dependent Variable: Carotid-femoral pulse wave velocity (m/s)

Supplemental file B:

Additional effects linear regression model with PWV as outcome variable (dependant variable).

Model Summary

	R	R Square	Adjusted R Square	Std. Error of the Estimate
Additional effects	.664[a]	0.441	0.362	0.83472

a. Predictors: Constant, smoker (yes), low-density lipoproteins (mmol/l), high sensitivity CRP (ng/l), estimated glomerular filtration rate (eGFR) (ml/min), age (years), Current tuberculous (yes), African (No), haematocrit (%), resting heart rate (beats/min), HIV infected (yes), mean blood pressure (mmHg), female sex (yes), body mass index (kg/m^2)

ANOVA[a]

	Sum of Squares	df	Mean Square	F	P value
Regression	50.510	13	3.885	5.576	.000[b]
Residual	64.102	92	0.697		
Total	114.612	105			

a. Dependent Variable: Carotid-femoral pulse wave velocity (m/s)

b. Predictors: Constant, smoker (yes), low-density lipoproteins (mmol/l), high sensitivity CRP (ng/l), estimated glomerular filtration rate (eGFR) (ml/min), age (years), current tuberculosis (yes), African (No), haematocrit (%), resting heart rate (beats/min), HIV infected (yes), mean blood pressure (mmHg), female sex (yes), body mass index (BMI) (kg/m^2)

Coefficients[a]

	Unstandardized Coefficients	Standardized Coefficients	P	95% Confidence Interval for B	
	B	Beta	value	Lower Bound	Upper Bound
Constant	2.491		0.082	-0.324	5.306
Age (years)	0.023	0.172	0.061	-0.001	0.048

Female sex (Yes)	-0.389	-0.187	0.078	-0.823	0.045
African (No)	-0.234	-0.101	0.277	-0.658	0.191
BMI (kg/m2)	0.020	0.110	0.415	-0.028	0.068
Mean blood Pressure (mmHg)	0.043	0.472	0.000	0.024	0.061
Resting heart rate (beats/min)	0.004	0.054	0.544	-0.009	0.017
eGFR (ml/min)	-0.017	-0.461	0.000	-0.026	-0.008
Haematocrit (%)	-0.119	-0.007	0.948	-3.756	3.517
Low density lipoproteins (mmol/l)	0.081	0.064	0.456	-0.133	0.294
High sensitivity CRP (ng/l)	-0.002	-0.042	0.623	-0.010	0.006
Current tuberculosis (Yes)	-0.250	-0.082	0.346	-0.775	0.275
HIV infected (Yes)	0.466	0.179	0.072	-0.043	0.976
Smoker (Yes)	0.145	0.069	0.486	-0.266	0.556

a. Dependent Variable: Carotid-femoral pulse wave velocity (m/s)

Supplemental file C:

Final linear regression model with PWV as outcome variable (dependant variable).

Model Summary

	R	R Square	Adjusted R Square	Std. Error of the Estimate
Final	.648[a]	0.419	0.390	0.81577

a. Predictors: Constant, estimated glomerular filtration rate (eGFR) (ml/min), age (years), female sex (yes), HIV infected (yes), mean blood pressure (mmHg)

ANOVA[a]

	Sum of Squares	df	Mean Square	F	P value
Regression	48.064	5	9.613	14.445	.000[b]
Residual	66.549	100	0.665		
Total	114.612	105			

a. Dependent Variable: Carotid-femoral pulse wave velocity (m/s)

b. Predictors: Constant, estimated glomerular filtration rate (eGFR) (ml/min), age (years), female sex (yes), HIV infected (yes), mean blood pressure (mmHg)

Coefficients[a]

	Unstandardized Coefficients	Standardized Coefficients	P	95% Confidence Interval for B	
	B	Beta	value	Lower Bound	Upper Bound
Constant	2.232		0.007	0.627	3.837
Age (years)	0.026	0.189	0.024	0.003	0.048
Female sex (yes)	-0.369	-0.177	0.024	-0.689	-0.049
HIV Infected (yes)	0.524	0.201	0.013	0.112	0.936
Mean blood	0.043	0.472	0.000	0.026	0.059

pressure (mmHg)					
eGFR (ml/min)	-0.015	-0.397	0.000	-0.021	-0.008

a. Dependent Variable: Carotid-femoral pulse wave velocity (m/s)

Note: The HIV infection coefficient is largely unchanged between models and remains a statistically significant contributor to PWV.

Supplemental file D:

Final linear regression model with log10 transformed aortic distensibility as outcome variable (dependant variable).

Model Summary

Model	R	R Square	Adjusted R Square	Std. Error of the Estimate
	.646[a]	0.417	0.388	0.13116

a. Predictors: Predictors: Constant, estimated glomerular filtration rate (eGFR) (ml/min), age (years), female sex (yes), HIV infected (yes), mean blood pressure (mmHg)

ANOVA[a]

Model	Sum of Squares	df	Mean Square	F	P value
Regression	1.233	5	0.247	14.334	.000[b]
Residual	1.720	100	0.017		
Total	2.953	105			

a. Dependent Variable: Log10 transformed aortic distensibility ($mmHg^{-1}$)

b. Predictors: Constant, estimated glomerular filtration rate (eGFR) (ml/min), age (years), female sex (yes), HIV infected (yes), mean blood pressure (mmHg)

Coefficients[a]

Model	Unstandardized Coefficients B	Standardized Coefficients Beta	P value	95% Confidence Interval for B Lower Bound	Upper Bound
Constant	0.092		0.480	-0.166	0.350
Age (years)	-0.004	-0.171	0.042	-0.007	0.000
Female sex	0.068	0.205	0.010	0.017	0.120
HIV infection	-0.090	-0.214	0.008	-0.156	-0.024
Mean blood pressure	-0.006	-0.435	0.000	-0.009	-0.004

(mmHg)					
eGFR (ml/min)	0.002	0.418	0.000	0.001	0.004

a. Dependent Variable: Log10 transformed aortic distensibility ($mmHg^{-1}$)
